# Pneumocephalus Following Combined Spinal Epidural Anaesthesia for Total Knee Arthroplasty: A Case Report

**DOI:** 10.5704/MOJ.1711.001

**Published:** 2017-11

**Authors:** YW Chew, VK Suppan, SR Ashutosh, MM Tew, JH Jimmy-Tan

**Affiliations:** Department of Orthopaedics, Hospital Sultan Abdul Halim, Sungai Petani, Malaysia; ^*^Department of Orthopaedics, Melaka Manipal Medical College, Bukit Baru, Malaysia; ^**^Clinical Research Centre, Hospital Sultan Abdul Halim, Sungai Petani, Malaysia; ^***^Department of Anaesthetic, Hospital Sultan Abdul Halim, Sungai Petani, Malaysia

**Keywords:** pneumocephalus, total knee arthroplasty, epidural anaesthesia, loss of resistance to air (LORA)

## Abstract

The authors describe a case of pneumocephalus following epidural anaesthesia for total knee arthroplasty. Multiple attempts in locating the epidural space for the anaesthesia and the use of loss of resistance to air (LORA) technique were identified as the source of air entry. Supportive management was given including high flow oxygenation therapy and spontaneous reabsorption of air was noted five days after surgery. The presence of pneumocephalus should be kept in mind if patient develops neurological complications postoperatively following epidural anaesthesia.

## Introduction

Lumbar epidural anaesthesia is a common technique used during lower limb surgery. Loss of resistance to air (LORA) technique is widely used for identification of epidural space with several complications reported such as pneumocephalus^1,2^, subcutaneous emphysema^3^, venous air embolism^4^, and spinal cord and nerve root compression^5^. We report an unusual case of pneumocephalus following epidural anaesthesia in a patient who underwent total knee arthroplasty.

## Case Report

A 75-year old gentleman with controlled, uncomplicated essential hypertension and ischemic heart disease under follow-up underwent elective total knee arthroplasty. He was on Tab. Clopidogrel 75mg OD for heart disease which was stopped one week prior to surgery. His coagulation tests on admission were within normal limits.

Combined spinal epidural (CSE) was chosen as the technique of anaesthesia and post operational analgesia for this patient. Patient’s back was prepared in the sitting position and draped with aseptic precautions. Patient vital signs were monitored. 2cc of 2% lidocaine was infiltrated for skin analgesia at L3/L4 interspace. B Braun CSE set was used. An 18G Tuohy needle was used to identify the epidural space midline approach using LORA technique. There was some difficulty in identifying the epidural space which required more than one attempt at L3/L4, L4/L5 with a total use of 2-3cc of air. No bleeding was noted. Single poke technique was used in this case. Skin to epidural space depth was 6cm and there did not appear to be any accidental puncture of dura. Standard procedure of 3cc of 0.5% hyperbaric bupivacaine alone was injected through spinal needle and epidural catheter was inserted and anchored at 10cm. There was no immediate complication.

**Fig. 1:**
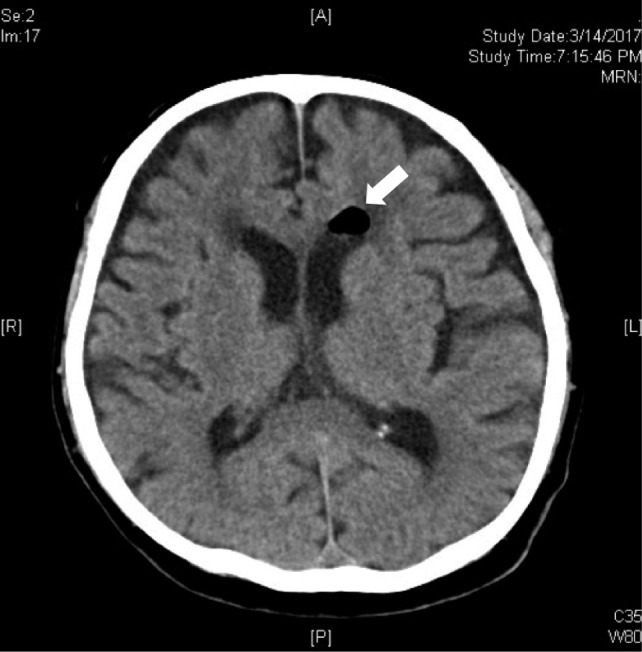
Emergency CT Brain within one hour post-seizure. Focal air pocket in the frontal horn of the left ventricle of the brain noted. (Shown by arrow)

**Fig. 2:**
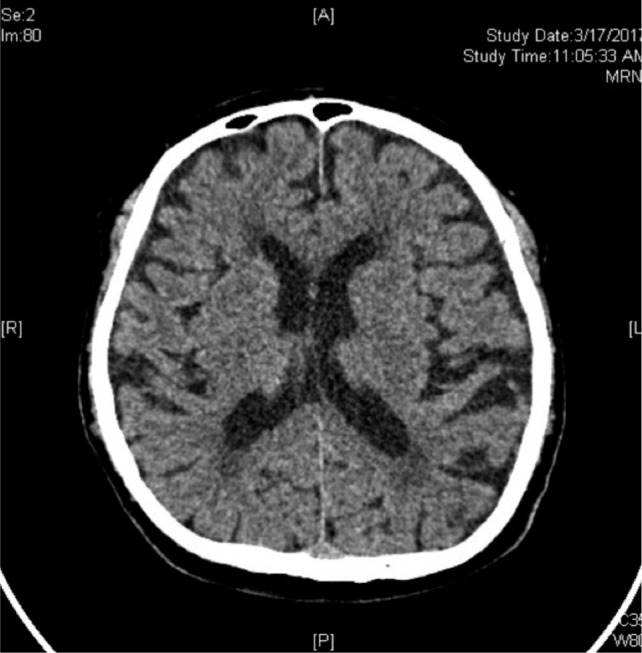
CT Brain repeated 3 days after the seizure showing complete resolution of the air pocket.

The surgical procedure was uneventful and lasted 50 minutes. Patient continued to remain stable in the recovery room and was transferred back to the general orthopaedic ward subsequently. The post operation epidural analgesics were given at infusion rate of 5cc/hour (the epidural cocktail was 1mg levobupivacaine and 2mcg fentanyl per cc). No complications were noted during the administration of the epidural analgesia. Post operational oral analgesic served in the ward included Tab Etoricoxib 120mg OD, Tab Paracetamol 1g TDS and IM Tramadol 50mg/ml PRN. Approximately 40 hours (day two) after surgery, the patient had a brief episode of unresponsiveness lasting a few seconds with up-rolling of eyeballs. The episode aborted spontaneously, followed by postictal drowsiness. Vital signs were within normal limits and there was no neurological deficit. Serum electrolytes and blood gases were within normal limits. No recent history of brain trauma or infection was reported and the blood sugar level was within normal range. Emergency computed tomography of brain (CT Brain) within an hour of seizure, revealed focal air pocket (size of the air pocket = 13.05mm x 10.12mm) in frontal horn of left ventricle of brain (Fig. 1). Epidural catheter was removed 48 hours after surgery. Patient was transferred to intensive care ward for close observation and supportive management. He showed no signs of Post-dural Puncture Headache (PDPH) nor did he have any subsequent convulsion episode. A repeat CT brain performed three days later showed complete resolution of air pocket (Fig. 2). He remained in hospital for a total period of five days after surgery.

He attended follow-up clinics and remained symptom free and comfortable.

## Discussion

Pneumocephalus is defined as presence of air or gas within the cranial cavity. The most common cause for pneumocephalus is trauma^1^. Other causes have been reported such as barotrauma, spinal surgery, instrumentations and in rare cases of infections. During literature search we did not notice pneumocephalus observed after spinal or epidural anaesthesia in orthopaedic-related surgery.

Pneumocephalus can be classified by location as extra-axial, comprising of epidural, subdural and subarachnoid and intra-axial which are parenchymal, intra-ventricular and intravascular. In our case, pneumocephalus developed probably due to air injected into the epidural space during multiple attempts of locating the space. Commonly, pneumocephalus is asymptomatic, however tension pneumocephalus can result in headache and features of increased intracranial tension.

Several case reports noted headache and dizziness as the main complaints in patients suffering from post-spinal anaesthesia pneumocephalus^2^. Our patient presented with a brief episode of acute onset seizure which lasted less than two minutes more than 36 hours post-surgery. He remained clinically well and free of complaints prior to and after the episode.

Diagnosis of pneumocephalus can be made with radiography, CT or MRI of brain. CT scan is highly sensitive as a specific diagnostic tool and can detect as little as 0.55ml of air within the cranial vault. In our case emergency CT brain showed a focal air pocket at the frontal horn of left lateral ventricle explaining the presentation.

Nistal and Gomez postulated mechanism of pneumocephalus as being due to dural puncture when a catheter goes into the subarachnoid space after its initial subdural placement and movement to the arachnoid and the first puncture in the dura yields migration of catheter^5^. Air introduced into subdural space is more painful than in subarachnoid space and reaches the head rapidly due to low pressure and diminished capacitance^4^. Our patient had no symptoms of headache and dizziness to indicate tension pneumocephalus.

Gas or air in brain following pneumocephalus gets absorbed and conservative management includes placing patient in Fowler position of 30°, avoiding Valsalva manoeuvre, management of pain and pyrexia to prevent hyperthermia and use of osmotic diuretics. Reabsorption is observed in 85% of cases in 2-3 weeks. In the absence of reabsorption, hyperbaric oxygenation therapy is useful, as demonstrated by Paiva *et al*^3^. In cases of tension pneumocephalus, treatment options include burr holes, needle aspiration and closure of dural defect^4^. Our patient was placed in the Fowler position and provided supportive therapy. Spontaneous reabsorption occurred in four days as confirmed by repeat CT brain. Patient remained symptom free subsequently.

Nistal and Gomez suggested minimization in the amount of air used in identification of epidural space in LORA technique to lower risk of pneumocephalus. Saline instead of air had been recommended alternatively to avoid pneumocephalus^5^.

## Conclusion

Pneumocephalus following epidural analgesia is not uncommon and may occur in spinal and cranial surgery as well as during labour. However this is probably the first case in CSE for total knee arthroplasty. Presence of pneumocephalus should be kept in mind if patient develops neurological complications post-surgery involving epidural anaesthesia. High degree of clinical suspicion and early recognition are important in preventing chronic complications as neuraxial anaesthesia is getting more popular and favourable as compared to general anaesthesia in total knee arthroplasty. We attribute the complication to be a problem of technique, however, an accidental small perforation of the dura cannot be ruled out. Loss of resistance using saline (LORS) has lower incidence of pneumocephalus and minimal amount of air should be used while using LORA technique to reduce the incidence of pneumocephalus.

This case report offers guidance and raises awareness of the incidence and management of pneumocephalus. The use of saline instead of air should be considered in epidural anaesthesia to minimise incidence of pneumocephalus^5^.
